# History of mild traumatic brain injury is associated with deficits in relational memory, reduced hippocampal volume, and less neural activity later in life

**DOI:** 10.3389/fnagi.2013.00041

**Published:** 2013-08-22

**Authors:** Jim M. Monti, Michelle W. Voss, Ari Pence, Edward McAuley, Arthur F. Kramer, Neal J. Cohen

**Affiliations:** ^1^Department of Psychology, University of Illinois at Urbana ChampaignChampaign, IL, USA; ^2^Beckman Institute, University of Illinois at Urbana ChampaignUrbana, IL, USA; ^3^Department of Psychology, University of IowaIowa City, IA, USA; ^4^Department of Kinesiology and Community Health, University of Illinois at Urbana ChampaignUrbana, IL, USA; ^5^Neuroscience Program, University of Illinois at Urbana ChampaignUrbana, IL, USA

**Keywords:** mTBI, hippocampus, aging, relational memory, fMRI

## Abstract

Evidence suggests that a history of head trauma is associated with memory deficits later in life. The majority of previous research has focused on moderate-to-severe traumatic brain injury (TBI), but recent evidence suggests that even a mild TBI (mTBI) can interact with the aging process and produce reductions in memory performance. This study examined the association of mTBI with memory and the brain by comparing young and middle-aged adults who have had mTBI in their recent (several years ago) and remote (several decades ago) past, respectively, with control subjects on a face-scene relational memory paradigm while they underwent functional magnetic resonance imaging (fMRI). Hippocampal volumes were also examined from high-resolution structural images. Results indicated middle-aged adults with a head injury in their remote past had impaired memory compared to gender, age, and education matched control participants, consistent with previous results in the study of memory, aging, and TBI. The present findings extended previous results by demonstrating that these individuals also had smaller bilateral hippocampi, and had reduced neural activity during memory performance in cortical regions important for memory retrieval. These results indicate that a history of mTBI may be one of the many factors that negatively influence cognitive and brain health in aging.

## Introduction

A hallmark of cognitive aging is inter-individual variability, with some individuals in their seventh and eighth decades already experiencing Alzheimer's disease while others continue to be productive in their careers. Explaining this variability is a critical challenge for the field of cognitive aging. Understanding and predicting one's cognitive phenotype in middle-to-late adulthood is difficult due to the myriad of interacting factors that influence cognitive aging. The relationship between cognition and age is not a simple linear reflection of the number of years lived; rather, it is a complex process influenced by the passage of time, inherent, and acquired neuroprotective factors (e.g., genetics and aerobic exercise), and the accumulation of events and processes with deleterious impacts on brain health, such as oxidative stress and hypertension (Mesulam, 2000; Fotuhi et al., 2009). An important factor thought to affect brain and cognitive aging that we know relatively little about are the effects of a history of traumatic brain injury (TBI), as this may interact with aging processes to produce poorer outcomes (Moretti et al., 2012).

Research at the intersection of TBI and aging has historically focused on either the effects of repetitive head trauma, or how moderate-to-severe TBI [such as penetrating head injuries or injuries resulting in loss of consciousness (LOC) greater than 30 min], influence an individual's cognitive aging trajectory. For instance, it is well-known that repeated head trauma, such as the type accumulated by a professional boxer, can lead to dementia later in life (Martland, 1928). More contemporary research has revealed that sustaining even a single moderate-to-severe TBI has negative implications on brain and cognition with advancing age. Research from Corkin et al. (1989) showed that those who suffered a penetrating brain injury in young adulthood displayed exacerbated cognitive decline with aging in a variety of cognitive domains, suggesting an interplay between early head trauma and the aging process. Complimentary evidence from neuropathological data further bolsters the claim that a history of head trauma is associated with worse outcomes in brain health with advancing age. Johnson and colleagues quantified the amount of neurofibrillary tangles (NFTs) and amyloid-beta (Aβ) plaques in survivors of a TBI after they died years later of causes unrelated to the injury (Johnson et al., 2012). Compared to controls, those who had a TBI in their life displayed more widespread amyloid pathology that was fibrillary in nature, as well as a greater deposition of NFTs in patients under the age of 60. These data are consonant with epidemiological studies indicating moderate-to-severe TBI leads to an increased risk or the hastening of developing Alzheimer's disease (Sullivan et al., 1987; Plassman et al., 2000).

Only recently has attention shifted to whether cognitive aging is affected by mTBI or concussion, which is when a kinetic force to the head or body (causing the head to rapidly accelerate and decelerate) results in brief or no LOC, and cognitive deficits that seemingly resolve over the short term. Of the 1.7 million TBIs that occur annually, 75% are classified as mild TBI (mTBI; Centers for Disease Control, 2003; Faul et al., 2010). Given the high prevalence of mTBI compared to moderate or severe TBI, investigation of how a history of mTBI impacts brain and cognitive health later in life may prove critical to understanding individual differences in cognitive aging. One line of studies investigating the effect of distal mTBI on cognitive aging examined former collegiate athletes that sustained mTBI's in college ~30 years earlier. Results showed deficits in declarative memory using neuropsychological tests when measured several decades after mTBI (De Beaumont et al., 2009; Tremblay et al., 2013). Compared to former college athletes without a history of mTBI, those who had sustained mTBI during their college career showed deficits in figural memory tasks, such as the Rey–Osterrieth Complex Figure. The poorer memory function was accompanied by metabolic abnormalities within the medial temporal lobe (MTL) of the group with a history of mTBI, as well as greater cortical thinning in frontal, parietal, and temporal cortices, compared to the control group (Tremblay et al., 2013). Taken together, these studies indicate prior history of mTBI may exacerbate and/or accelerate age-related memory decrements and neural changes.

The current study examined the possible interaction of age and mTBI, combining behavioral assessment of relational (declarative) memory with structural and functional assessment of the hippocampus and the cortical regions with which it interacts. The focus here was on the hippocampal memory system (Cohen and Eichenbaum, 1993; Eichenbaum and Cohen, 2001), based on converging evidence from several lines of work. The hippocampus has been shown to be greatly affected not only by age-related diseases (e.g., Alzheimer's disease, vascular dementia) but also by disease-free aging or so-called “healthy aging” (Small et al., 2011; Jagust, 2013). Longitudinal studies have consistently demonstrated hippocampal volumetric declines in older adults (Raz et al., 2005, 2010). Additionally, even in the absence of Alzheimer's disease, tau pathology is ubiquitously found in the entorhinal cortex and hippocampus in older adults (Price et al., 2009; Bennett et al., 2012), and the presence of this pathology relates negatively to memory performance (Guillozet et al., 2003; Bennett et al., 2012). Moreover, changes in basal metabolism occur within the hippocampus with aging and age-related diseases (for review, see Small et al., 2011).

Further, there is reason to believe the hippocampus is disproportionately affected by TBI; its physical location in the MTL makes it more vulnerable to impact forces, and it is also particularly susceptible to excitotoxic injury, which occurs in TBI (McAllister, 2011). Several MR studies in moderate-to-severe TBI populations have indicated significantly smaller hippocampal volumes within 2 years of the initial injury (Bigler et al., 2002; Hopkins et al., 2005; Ariza et al., 2006). A study of pediatric TBI found reduced hippocampal and amygdala volumes at 10-year follow-up, which was true even for a group only sustaining mTBI (Beauchamp et al., 2011). Finally, animal models of mTBI reveal large apoptotic changes in the CA3 subfield of the hippocampus following experimenter-induced mTBI, second only to the anterior cingulate gyrus in magnitude of neuronal damage (Tashlykov et al., 2007). Taken altogether, the hippocampus would appear to be a particularly suitable target for detecting negative changes elicited by the combination of prior mTBI and aging. Here its status was assessed structurally, via measurement of volume of the hippocampus in comparison to other subcortical structures, and functionally, via examination of brain activation and memory performance during mnemonic challenge using functional magnetic resonance imaging (fMRI).

Functional assessment focused on relational memory, i.e., the ability to acquire and retain memory for arbitrary or accidental relations amongst the constituent elements of a scene, event, or experimental session (Cohen and Eichenbaum, 1993; Eichenbaum and Cohen, 2001). Relational memory is critically dependent on the hippocampus (Ryan et al., 2000; Hannula et al., 2006, 2007; Konkel et al., 2008; Watson et al., 2013), and its successful expression engages the hippocampus and larger brain networks involving MTL-cortical (perirhinal, entorhinal, and parahippocampal cortex) and neocortical (principally, prefrontal and posterior parietal) regions (Cohen et al., 1999; Simons and Spiers, 2003; Wagner et al., 2005; Cabeza et al., 2008; Staresina and Davachi, 2009). The dependence of relational memory on both the hippocampus and large-scale brain networks make it a favorable target for the current investigation, with the prediction that individuals who had sustained mTBI several decades ago would show decrement in this form of memory and would show structural and functional brain aberrations in the brain regions and networks subserving relational memory.

## Materials and methods

### Participants

Participants were administered a standardized questionnaire to ascertain if they had a history of mTBI. This questionnaire gathered information regarding the number of head injuries, approximate date(s), description of the event(s), and duration of symptoms (including LOC and duration, confusion/disorientation, length of post-traumatic amnesia). Only those participants whose mTBIs were diagnosed by a medical professional and/or resulted in LOC were included in the mTBI groups. Additionally, in order to ensure all TBIs were categorized as “mild,” any individuals who experienced a LOC greater than 30 min or post-traumatic amnesia longer than 24 h were excluded from this study (American Congress of Rehabilitation Medicine, 1993). Finally, participants suffering an mTBI after the age of 25 were excluded from the study, as to isolate the combined effects of early mTBI and the aging process. A total of 22 participants met these criteria. For comparison, 22 age, gender, and education matched participants without a history of major head trauma were also included. All 44 participants in the final sample were screened for, and did not report, any alcohol abuse. Participants were categorized into two groups based on current age, with ages 20–29 years being classified as “young” and 40–69 years as “middle-aged.” This yielded four experimental groups: young control (YC), young mTBI (YI), middle-aged control (MC), and middle-aged mTBI (MI; see Table 1). The two mTBI groups reported similar durations of LOC, which for both groups ranged from a few seconds to approximately five minutes, with one exception in the YI group of an individual reporting a duration of ~20 min. This study was approved by the University of Illinois Institutional Review Board, and all participants signed an informed consent document.

**Table 1:** **Participant demographics and memory performance: mean (SD)**.

**Variable**	**YC**	**YI**	**MC**	**MI**
*N*	12 (5 female)	12 (5 female)	10 (6 female)	10 (6 female)
Age	22.3 (2.6)	22.4 (2)	52.5 (7.9)	52.9 (9.4)
Years of education	15.6 (0.9)	15.4 (0.52)	17.3 (3.6)	17.3 (3.9)
Number of mTBIs	–	1.3 (0.62)	–	1.4 (0.52)
Years since last mTBI	–	4 (3.1)	–	39 (12.6)
LOC	–	10	–	9
Hit rate	0.81 (0.18)	0.84 (0.08)	0.85 (0.1)	0.73 (0.13)
False alarm rate	0.28 (0.14)	0.25 (0.13)	0.33 (0.14)	0.33 (0.12)

mTBI, mild traumatic brain injury; LOC, loss of consciousness; YC, young control group; YI, young mTBI group; MC, middle-aged control group; MI, middle-aged mTBI group. Number of mTBIs refers to group average, the range for the YI group was 1–3, and the MI group 1–2. LOC identifies the number of participants who sustained an mTBI that resulted in LOC less than 30 min in duration. Hit rate and false alarm rate pertain to performance on the memory task.

### Memory task

**Figure 1:**
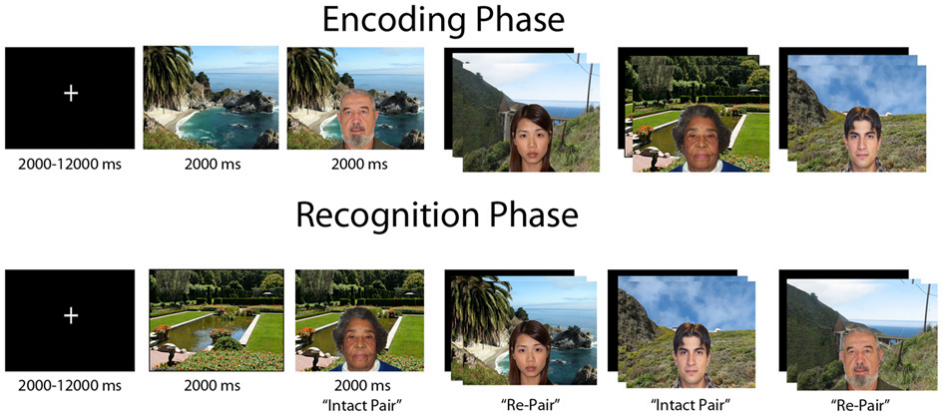
**Figural depiction of encoding and recognition phases of relational memory task**. Each trial consisted of a jittered inter-trial-interval, followed by a scene preview and then the face overlaid on the scene. In a block, each phase consisted of 24 trials, with a 20 s break in between encoding and recognition. Participants completed three blocks of the task.

Participants completed an event-related relational memory task during fMRI scanning where the goal was to form an association between a face and scene (Figure 1). The task consisted of 72 images of outdoor scenes and 72 images of faces, of which 36 were male and 36 were female. Further, 36 of the faces were of young people and the other 36 of elderly people. The task was divided into three separate runs, with 24 encoding and 24 recognition trials in each run; the encoding and recognition phases were separated by a 20-s break. Encoding and recognition trials consisted of the presentation of a scene for 2000 millisec (ms), followed by a face overlaid on the scene for an additional 2000 ms. A fixation cross was displayed during the intertrial interval (ITI), which was jittered and ranged from 2000 to 12000 ms.

On each encoding trial, participants made a yes/no judgment indicating whether the individual depicted “fit” with the scene; this was an arbitrary decision to elicit deep encoding. At recognition two trial types were presented, “intact” face-scene pairs, which were the identical face-scene combinations presented during encoding, and “re-pair” trials created by recombining a previously displayed face and scene that were not shown together at encoding. Hence, all stimuli were equally familiar at recognition, and the task had to be completed via relational memory. Participants made a yes/no judgment as to whether the pair displayed was an exact match of a pair shown at encoding, with 12 trials from each trial type composing the recognition phase of a run.

A correct response to an intact pair was classified as a “hit,” with an incorrect response categorized as a “miss;” a correct response to a re-pair trial was a “correct rejection,” whereas an incorrect response was a “false alarm (FA).” A hit rate was derived for each participant by calculating the proportion of hits on trials with intact pairs, and a FA rate was obtained for each participant by finding the proportion of FAs on re-pair trials. Both the hit rate and FA rate were entered into 2 × 2 fixed effects ANOVAs with the factors of Age (young vs. middle-aged) and TBI history (control vs. mTBI).

### Image acquisition

All images were collected on a Siemens Trio 3-Tesla full body magnet, using a 12-channel birdcage headcoil. Functional BOLD images were acquired parallel to the anterior commissure-posterior commissure (AC-PC) line with a T2^*^-weighted echo-planar imaging sequence of 35 contiguous axial slices collected in ascending fashion [repetition time (TR) = 2000 ms; echo time (TE) = 25 ms; BOLD volumes = 299; flip angle = 80°; field of view (FOV) = 220 × 220 mm; voxel size = 3.4 × 3.4 × 4 mm]. Structural images were acquired with a T1-weighted 3D magnetization prepared rapid gradient echo imaging (MPRAGE) protocol of 192 contiguous sagittal slices collected in an ascending manner parallel to the AC-PC line [TR = 1900 ms; TE = 2.26 ms; flip angle = 9°; FOV = 256 × 256 mm; voxel size = 1 × 1 × 1 mm].

### Subcortical volume analysis

Automated segmentation of subcortical regions was conducted using Freesurfer (v 5.1); details of the subcortical segmentation process utilized by Freesurfer are available in Fischl et al. (2002). The algorithm employed by Freesurfer for subcortical segmentation has been shown to have a very high correlation with manual tracing, particularly for the hippocampus, and has proven to be sensitive to volume differences between groups (Morey et al., 2009). The main regions of interest (ROIs) for which volumetric data was estimated were the left and right hippocampus; to assess the specificity of the effect of mTBI and aging on hippocampal volume, bilateral putamen, caudate, and thalamus volumes were also estimated. An automated measure of intracranial volume (ICV), which is comparable to manual tracing, was obtained for each participant via Freesurfer using the methods described in Buckner et al. (2004). This measure of estimated ICV was used to correct subcortical volume for head size by regressing each ROI volume onto ICV in order to obtain a slope (*b*) for the relationship between an ROI and ICV. The resulting slope was then used to normalize each ROI for head size via the following formula: normalized volume = raw volume - *b* (ICV – mean ICV); this correction has been used in multiple studies reporting subcortical volume measures (Raz et al., 2005; Head et al., 2008; Erickson et al., 2009). The normalized volumes for each ROI were then entered into 2 × 2 ANOVAs with Age and TBI history as factors.

### Functional analysis

Preprocessing of the fMRI data was done using FSL 4.1.3 (FMRIB's Software Library, www.fmrib.ox.ac.uk/fsl). The first three volumes of each run were removed prior to analysis. For each run of functional data, a 100-s temporal high pass filter was applied, and data were smoothed with a Gaussian kernel of full width at half-maximum at 6 mm^3^. Also, motion correction was applied using MCFLIRT. Non-brain structures from the structural scan were removed and the image was co-registered to the subject's mean functional scans and placed into Montreal Neurological Institute (MNI) template space.

Each participant's three runs of the memory task were analyzed individually using FSL's FEAT (version 5.98) function. The hemodynamic response was convolved with a double-gamma HRF function. The statistical model for the task contained three regressors from the encoding phase, corresponding to whether a face-scene pair was later correctly remembered, forgotten, or went on to be a re-pair trial, and then five regressors in the recognition phase, with four coding the outcome of a trial (hit, miss, FA, correct rejection) and a nuisance variable tracking trials that were not responded to. All eight regressors corresponded to the onset of the scene and had a duration of 4 s. This model was then regressed against the observed fMRI data from each run. Analysis of each run consisted of creating contrasts from the events described above. Next, each contrast was averaged across an individual's three runs via a fixed effects analysis. Finally, group-level, mixed-effects analyses were conducted on data from the recognition phase, specifically looking at the contrasts of hits > correct rejections, hits > baseline, and correct rejections > baseline; these analyses were carried out using FSL's FLAME function with the variances for the two age groups estimated separately (Beckmann et al., 2003). The decision to investigate group differences for hits and correct rejections compared to baseline rather than relying solely on the hits > correct rejections contrast was due to both of these conditions likely having a high memory demand, given that the stimuli composing the foils at recognition have also been previously seen in the encoding phase; thus, it is possible that the hits > correct rejections contrast may remove a substantial amount of the BOLD signal in each individual, potentially masking group differences in neural processing. Therefore, the additional contrasts against baseline were also included for group comparison. The group level comparisons of the fMRI data were conducted via a 2 × 2 mixed effects ANOVA that considered between-subjects variance and contained the factors “Age” and “TBI history.” Significant main effects or an interaction were followed up with pairwise group comparisons. Reported functional imaging findings are significant at a voxel activation threshold of *Z* > 1.96 (*p* < 0.05), and a family wise error cluster correction threshold of *p* < 0.05.

## Results

**Figure 2:**
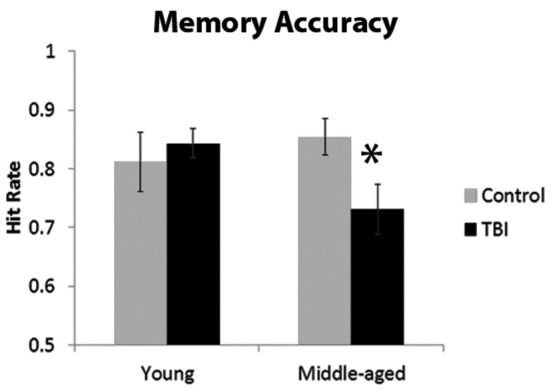
Behavioral data from memory paradigm, ^*^p < 0.05. Error bars are ± one standard error of the mean.

The ANOVA investigating the proportion of hits indicated a significant interaction with age and TBI history [*F*_(1, 40)_ = 3.93, *p* = 0.05]. Planned comparisons revealed that this interaction was driven by the MI group having a significantly lower hit rate than the MC group, [*t*_(18)_ = 2.37, *p* = 0.03], whereas there was no difference between the young groups [*t*_(22)_ = 0.56, *p* = 0.58; Figure 2]. There were no main effects of Age or TBI history for hit rate (all *F*'*s* < 1.4, *p*'*s* > 0.24). The FA rate data indicated the observed effect was not due to differences in response bias between the middle-aged groups, as analysis of the FA rate data did not reveal an effect of TBI history [*F*_(1, 40)_ = 0.08, *p* = 0.78] nor an Age × TBI history interaction [*F*_(1, 40)_ = 0.12, *p* = 0.73; see Table 1 for values]. Finally, there was no difference between age groups in the FA rate, [*F*_(1, 40)_ = 2.45, *p* = 0.13].

**Figure 3:**
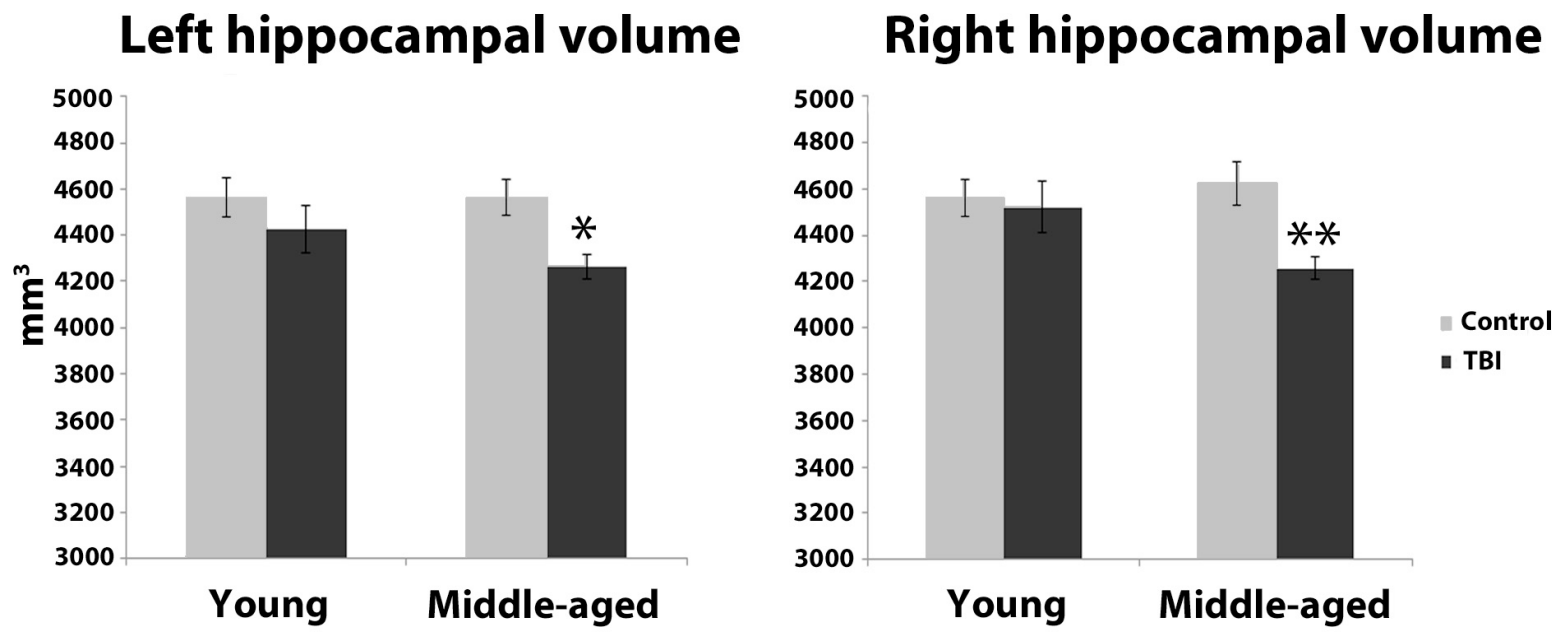
**Hippocampal volume differences as a function of the combination of distal mTBI and aging**. ^*^p < 0.05, ^**^p < 0.01; error bars are ± one standard error of the mean.

Estimation of hippocampal volume revealed main effects of TBI history for bilateral hippocampus, whereby the mTBI group had smaller hippocampal volumes: left hippocampus [*F*_(1, 40)_ = 7.18, *p* = 0.01]; right hippocampus [*F*_(1, 40)_ = 5.29, *p* = 0.03; see Table 2 for mean volumes]. In the right hippocampus there was a marginal Age × TBI history interaction, [*F*_(1, 40)_ = 3.42, *p* = 0.07]; this was not true of the left hippocampus, [*F*_(1, 40)_ = 1.00, *p* = 0.32]. Also, there was no main effect of Age in the left or right hippocampus (all *F*'*s* < 1.4, all *p*'*s* > 0.25). To ascertain if the marginal interaction in the right hippocampus, and the main effects that occurred bilaterally, were due to the hypothesized combination of aging and mTBI, planned comparisons were performed contrasting the TBI history factor at each level of Age. For the right hippocampus, the observed effects were due to differences between the two middle-aged groups. The MI group had a significantly smaller right hippocampus compared to the MC group [*t*_(18)_ = 2.97, *p* < 0.01], whereas there was no difference in hippocampus size between the two young groups [*t*_(22)_ = 0.32, *p* = 0.75]. Similarly, in the left hippocampus, a difference existed between the two middle-aged groups indicating smaller left hippocampus for the MI group, [*t*_(18)_ = 2.65, *p* = 0.02], but no difference between the young adults [*t*_(22)_ = 1.19, *p* = 0.25]. Hippocampal volume differences amongst the four groups are depicted graphically in Figure 3. The above finding of smaller subcortical tissue due to a combination of mTBI and aging seems specific to the hippocampus, as analyses of the left and right putamen, caudate, and thalamus did not show any effects of TBI history or interactions between Age and TBI history (all *F*'*s* < 1.4, all *p*'*s* > 0.24). There were large effects of Age for both the left [*F*_(1, 40)_ = 16.43, *p* < 0.001] and right putamen [*F*_(1, 40)_ = 17.37, *p* < 0.001], with the middle-aged groups having smaller volumes. There were trends for smaller left [*F*_(1, 40)_ = 3.34, *p* = 0.08] and right [*F*_(1, 40)_ = 3.53, *p* = 0.07] caudate nuclei in the middle-aged groups as well.

**Table 2:** **Subcortical volume estimates: mean (SD)**.

**Region**	**YC**	**YI**	**MC**	**MI**
Left hippocampus	4561 (295)	4424 (269)	4563 (321)	4263 (158)
Right hippocampus	4561 (283)	4521 (322)	4624 (353)	4258 (165)
Left putamen	6321 (579)	6132 (575)	5710 (278)	5552 (393)
Right putamen	5797 (572)	5657 (490)	5203 (455)	5059 (299)
Left caudate	3769 (420)	3878 (338)	3573 (502)	3621 (369)
Right caudate	3778 (394)	3843 (392)	3580 (431)	3604 (307)
Left thalamus	7690 (549)	7659 (534)	7682 (945)	7290 (493)
Right thalamus	7726 (523)	7691 (380)	7711 (606)	7454 (634)

YC, young control group; YI, young mTBI group; MC, middle-aged control group; MI, middle-aged mTBI group. Values are reported in mm^3^ and are corrected for intracranial volume.

**Figure 4:**
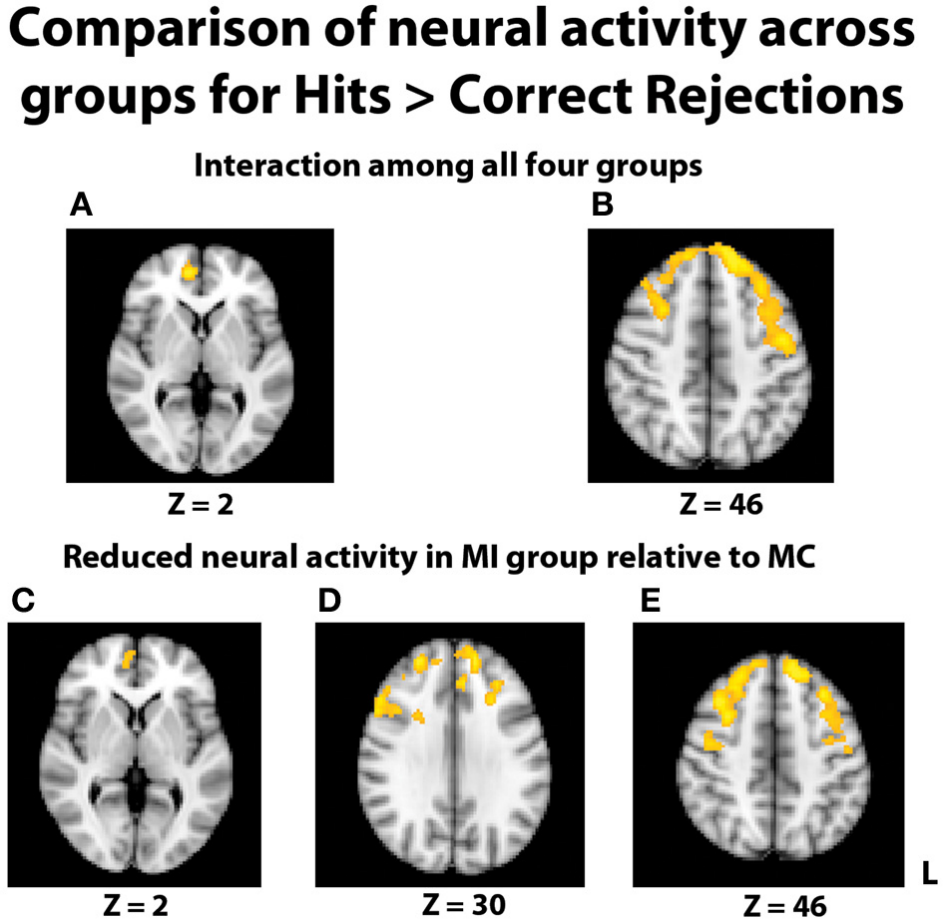
**Images displayed depict differential group activity for the contrast of hits > correct rejections**. **(A,B)** Brain regions significant for the interaction between age and TBI history, as measured by the contrast (YI + MC > YC + MI). Areas with significant activity include the right medial PFC, left precentral gyrus, and bilateral superior frontal gyri. **(C–E)** Reductions in neural activity for the MI group relative to the MC group in multiple areas of the PFC. Coordinates are in MNI space; images are in radiological orientation (L = R).

In the fMRI data, the group-level ANOVA on the contrast of hits > correct rejections at recognition revealed a significant interaction with activity in multiple areas of the prefrontal cortex (Figures 4A,B). To determine if this interaction was due to differences specific to the middle-aged group, consistent with our hypothesis, follow-up contrasts were formed comparing TBI history at each level of Age. The contrast of brain activation between the young adult groups yielded no significant effects in either direction. However, reduced neural activity in multiple regions of the PFC was found for the MI group relative to the MC group (Figures 4C–E), including the right inferior frontal gyrus, right medial PFC (mPFC), bilateral frontopolar cortex, and middle and superior frontal gyri. Additional pairwise comparisons revealed that this interaction was also due in part to the YI group having more activity than the MI group in PFC and parietal regions.

**Figure 5:**
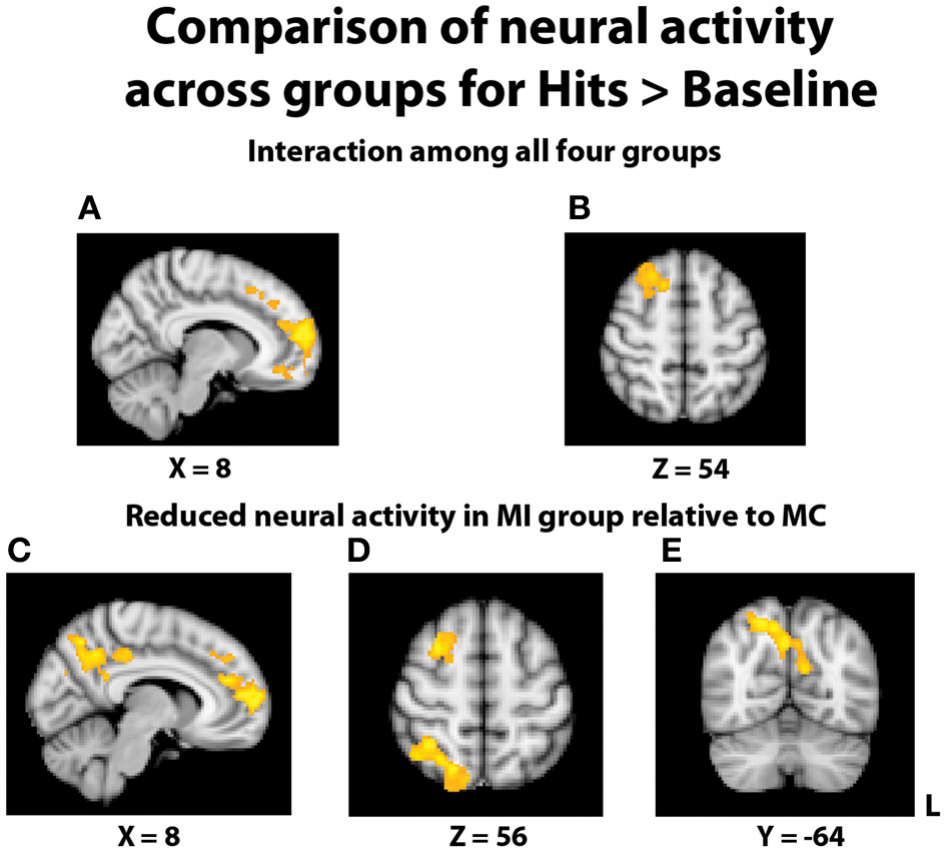
**Illustration of significant differential group activity for hits > baseline**. **(A,B)** Significant activity for the interaction of age and TBI history, as measured by the contrast (YI + MC > YC + MI). Areas of significant activation included right medial prefrontal cortex and right superior frontal gyrus. **(C–E)** Reductions in neural activity for the MI group relative to the MC group were found in medial and lateral PFC, right superior parietal lobe, right posterior cingulate cortex, and bilateral precuneus. Coordinates are in MNI space; images are in radiological orientation (L = R).

In an effort to more fully understand any group differences in the BOLD response during relational memory recognition, each participant's contrasts of hits > baseline and correct rejections > baseline were analyzed at the group level. With regard to the analysis of hits > baseline, once again a significant interaction indicated a differential BOLD signal between the groups in the PFC (Figures 5A,B). In similar fashion to the hits > correct rejection analysis, pairwise comparisons were conducted between the TBI groups at both levels of Age. There were no significant differences between the two young groups, but the comparison of the middle-aged participants again revealed areas of reduced neural activity in the MI group. Multiple regions of differential activation were found in the PFC, localized within the right mPFC, frontal pole, paracingulate gyrus, right middle and superior frontal gyri, and left superior frontal gyrus. Regions in the parietal lobe also displayed reductions in activity for the MI group, with a large swath of activity in medial parietal regions including bilateral precuneus and right posterior cingulate cortex, and another area found in the right superior parietal lobule (SPL), extending into the parieto-occipital junction (Figures 5C–E). Further pairwise comparisons indicated the observed interaction was also due to the MC group displaying more activity than the YC group in the frontal cortex and left lateral and parietal lobes.

The comparison of correct rejections > baseline did not yield any differential activity related to TBI status among the groups; this indicates that the differences due to TBI group status in the hits > baseline comparison seem to be due to successful memory recognition and not group differences in perception or attention, which is possible due to the baseline comparison. Finally, in all of the above comparisons, there were no brain regions where the MI group had greater activity than the MC group.

Given the differing levels of accuracy between the two groups, it is conceivable that the fMRI differences in activation are due to the performance differences; we believe this is unlikely for two reasons. First, while the difference in the hit rate was significant, the average number of additional trials analyzed in the fMRI data for the MC group was 4.3 out of a possible 36; this modest disparity in trial count reduces the chance that the activation differences are due to performance. Additionally, percent signal change for each participant was extracted from distinct anatomical areas of peak activation for the contrasts of hits > CR and hits > baseline, and then correlated with performance. Correlations were carried out separately for each group, as to avoid circular analyses. Regions of peak activation for hits > CR included: left superior frontal gyrus, right superior frontal gyrus, right middle frontal gyrus, and right mPFC, while regions for hits > baseline were: right precuneus, right SPL, right paracingulate cortex, right middle frontal gyrus, and right frontal pole. Of the 18 correlations, only two were significant at *p* < 0.05, the right middle frontal gyrus for hits > CR in the MI group, and the right SPL in hits > baseline for the MC group. Therefore, the differences in the BOLD response between the two middle-aged groups seem to reflect reduced neural activity and/or differential neural processing in the MI group rather than a simple lack of power or reflection of performance differences in the MI group.

## Discussion

Compared to age- and education-matched controls with no history of head trauma (MC group), those with an mTBI decades ago (MI group) were less accurate on a face-scene relational memory task, and had less neural activity for successful memory recognition in posterior parietal cortex and PFC. Moreover, the MI group had smaller hippocampi bilaterally than the MC group. There was no impairment evident in memory or differences apparent in neuroimaging in individuals with recent mTBI (YI group) relative to the neurologically intact comparison group, implicating the combination of aging and a history of mTBI as the source of the deficit observed in the MI group, rather than mTBI alone. Importantly, many of the individuals in our MI sample went on to attain a high level of education and achieved and maintained gainful employment despite their early mTBI. Thus, it does not appear to be the case that acute symptoms from their mTBI simply never resolved. Rather, it is more likely that the biological effects of aging that accrue over time and that are deleterious to brain health in all individuals serve to magnify the effects of early mTBI, eventually altering the cognitive phenotype and manifesting as impairment later in the course of aging.

The decreased accuracy in relational memory observed here for the MI group is consistent with previous studies investigating the effects of mTBI on memory in persons who sustained their mTBI several decades ago, including deficits in visuospatial or figural memory tasks, such as the Rey–Osterrieth Complex Figure (De Beaumont et al., 2009; Tremblay et al., 2013). This study expands the domain of memory related deficits to a non-visuospatial/figural memory task, here involving the remembering of arbitrary pairings of faces with scenes. Both the Rey–Osterrieth Complex Figure and memory for face-scene relations are known to be dependent on the hippocampus (Bohbot et al., 1998; Hannula et al., 2006, 2007; Hirni et al., 2013), suggesting the sensitivity of hippocampal-dependent memory processes to the effects of mTBI in interaction with aging.

The finding here of smaller hippocampal volumes bilaterally in the MI group compared to the MC group may be the major driving force of the observed memory differences. It is becoming increasingly clear that TBI is detrimental to hippocampal structure. Relationships between moderate-to-severe TBI and reduced hippocampal volume have been found when measured within 2 years of the initial injury (Bigler et al., 2002; Hopkins et al., 2005; Ariza et al., 2006). One study of childhood TBI found hippocampal volumes to be reduced in a sample of mTBI patients at a 10-year-follow-up (Beauchamp et al., 2011). To our knowledge, though, this is the first study reporting smaller hippocampal volumes due to a combination of distal mTBI and aging in middle-aged-to-older adults. Animal models of mTBI indicate the hippocampus is particularly vulnerable to apoptotic changes following mTBI (Tashlykov et al., 2007). Moreover, mTBI leads to states of increased oxidative stress and neuroinflammation in the hippocampus, as well as disruption of calcium ion homeostasis resulting in an influx of Ca^2+^ (Giza and Hovda, 2001; Wu et al., 2007; Gatson et al., 2013) all of which are deleterious to hippocampal health (Ekdahl et al., 2003; Serrano and Klann, 2004; Foster, 2007). Given that processes like neuroinflammation impair neurogenesis, coupled with the recent finding of only modest declines in neurogenesis with aging in adult humans, it is possible that different neurogenesis rates in the two middle-aged groups contribute to the volumetric findings (Ekdahl et al., 2003; Spalding et al., 2013). Finally, tau pathology co-occurs with TBI (McKee et al., 2013), and given the predilection of tau for MTL regions with aging, it is possible this may play a role in the observed volume differences. Since these hypothesized mechanisms are secondary molecular processes of mTBI rather than a direct result of physical trauma due to the initial impact, it is perhaps not surprising that the hippocampus, known to be sensitive to these mechanisms, is the structure that is smaller in the MI group (for review of molecular mechanisms following TBI, see Walker and Tesco, 2013). In considering the current findings, there are a number of potential pathogenic processes of the initial mTBI may mildly injure the hippocampus, leaving it more vulnerable to the plethora of pathogenic processes that co-occur with aging and constitute a “second hit” to this structure; longitudinal data will be crucial in identifying within-person atrophy in this population.

If aging does cause a second hit to hippocampal volume, the absence of any differences observed here in hippocampal volumes between the MC and YC groups might be somewhat surprising. However, recent research indicates a period of relative stability in hippocampal volume from the third to fifth decades of life, with marked declines in hippocampal volume not beginning until around age 50 (Fjell et al., 2013). Since the mean age of our MC group was 52.5, a significant portion of the current MC sample may not yet be experiencing age-related hippocampal volume loss. Even in those who are experiencing age-related hippocampal volume loss, its effects may be mitigated by the high level of education in the middle-aged groups, as education has been shown to mediate the effects of age-related volume loss in the hippocampus (Noble et al., 2012). By contrast, the large effect of age on putamen volume observed here is consistent with a recent report indicating a linear decline in putamen volume across the lifespan (Fjell et al., 2013).

The current fMRI findings, showing less activity for the MI group in PFC and posterior parietal lobe, implicate potential cortical dysfunction, or cortical involvement in the observed memory impairment. The MTL and PFC share many direct and indirect connections, and it is hypothesized that the ventral and dorsal lateral PFC may serve both to specify the parameters of MTL-dependent memory search, as well as to monitor the products of MTL memory retrieval for their relevance to task goals (Dobbins et al., 2002; Simons and Spiers, 2003). Activity in the posterior parietal lobe during successful memory recognition is consistently observed across many paradigms (Wagner et al., 2005; Cabeza et al., 2008) and posterior parietal regions are also connected directly or indirectly to regions in the MTL (Rockland and Van Hoesen, 1999; Kobayashi and Amaral, 2003). Current accounts of this posterior parietal activation occurring with successful memory recognition indicate that these regions may be exerting their influence via modulating attention related to memory, or informing memory decisions by integrating or temporarily storing information (Wagner et al., 2005; Cabeza et al., 2008). Therefore, it is possible the memory deficit observed in the MI group also stems from an inadequacy of these PFC and posterior parietal regions to coordinate memory recognition with an already disadvantaged hippocampus.

It should be noted that the cortical areas where differences between the MC and MI groups emerged overlap largely with regions of the default mode network (DMN). Like the hippocampus, DMN regions are also disproportionately susceptible to the aging process, and display reduced activity and connectivity with aging (Andrews-Hanna et al., 2007; Damoiseaux et al., 2008). Interestingly, even when only considering cognitively normal older adults, these DMN areas are known to be the locations of the greatest Aβ aggregation, and this Aβ accumulation in these regions is detrimental to hippocampal-based memory (Sperling et al., 2009; Rentz et al., 2011; Kennedy et al., 2012). Animal and human research has established a firm link between Aβ and TBI (for review, see Johnson et al., 2010; Walker and Tesco, 2013), with even post-mortem samples of individuals suffering mTBI showing expression of the amyloid precursor protein (Blumbergs et al., 1995). Though speculative, it may prove fruitful to examine whether a history of mTBI may help to explain the variability in cognitively normal middle-aged-to-older adults who harbor Aβ in the cortex (Kennedy et al., 2012).

There are some caveats to acknowledge. This was a cross-sectional study, and although the mTBI and control groups were matched on various factors, it is possible that they had additional differences other than their head injury; longitudinal studies will prove crucial to fully determining the nature of within subject change emerging from the combinatorial effects of mTBI and aging. Additionally, given the relatively small sample size and the relatively wide age range comprising the middle-aged group, some caution may be warranted in interpreting these results; but it is noteworthy that a similar study with a comparable sample size of middle-aged adults has reported complimentary findings (Tremblay et al., 2013).

Summarizing the findings of the current work, this report corroborates the memory deficit noted in previous studies on mTBI and aging, and includes the critical control of having both younger and older adults with and without a history of mTBI in order to document the interaction of prior mTBI and aging. Furthermore, it provides the first demonstration of reduced hippocampal size in individuals examined several decades following mTBI. Finally, the fMRI findings, revealing reductions in activity in posterior parietal cortex and PFC while performing a hippocampal-based memory task, provide evidence of global brain changes of mTBI in combination with the effects of aging. These data taken altogether make the case that a history of mTBI early in life may be an influencing factor on cognitive and brain health in older adulthood.

### Conflict of interest statement

The authors declare that the research was conducted in the absence of any commercial or financial relationships that could be construed as a potential conflict of interest.
